# Glucose priming effect on microbial intercellular metabolic flux diversity in a marine intertidal sediment

**DOI:** 10.1371/journal.pone.0335053

**Published:** 2025-11-26

**Authors:** Tao Ji, Zhiao Xu, Lihong Wen, Yuxue Yang, Yue Wu, Xueqin Zhao, Ruilian Yao, Rulong Liu, Yunping Xu, Weichao Wu

**Affiliations:** 1 College of Oceanography and Ecological Science, Shanghai Ocean University, Shanghai, China; 2 International Research Center for Marine Biosciences at Shanghai Ocean University, Ministry of Science and Technology, Shanghai, China; 3 State Key Laboratory of Microbial Metabolism, and School of Life Sciences and Biotechnology, Shanghai Jiao Tong University, Shanghai, China; University of Westminster - Regent Street Campus: University of Westminster, UNITED KINGDOM OF GREAT BRITAIN AND NORTHERN IRELAND

## Abstract

Microbial communities inhabiting intertidal sediment show diverse metabolisms to adapt to hydrodynamic conditions. However, the priming effect of exogenous organic matter on microbial metabolic fluxes remains poorly understood. Here we investigated microbial intercellular activities in surface sandy intertidal sediment (0–5 cm depth, from the Nanhui tidal flat, East China Sea) under anoxic conditions by incubating with position-specific ^13^C labelled glucose isotopologues. Elevated production of ^13^C-CO_2_ and phospholipid-derived fatty acids (PLFAs) was observed after 96-hour incubation, suggesting a positive priming effect on both catabolic and anabolic activities. The different incorporations of ^13^C-label from glucose isotopologues into CO_2_ and PLFAs reveal distinct intracellular carbon fluxes through the central metabolic network. Specifically, microbial communities preferentially utilize the Embden-Meyerhof-Parnas (EMP) pathway (62% flux), followed by the Pentose Phosphate (PP, 25%) and Entner-Doudoroff (ED, 13%) pathways. These flux distributions closely mirror their corresponding functional genome proportion (55% EMP, 26% PP, 20% ED), suggesting a metabolic balance between energy yield and enzyme cost across glycolytic routes. Furthermore, metabolic flux analysis based on ^13^C-PLFA profiles highlights diverse metabolic strategies among different microbial taxa, primarily involving the EMP, ED and tricarboxylic acid pathways. This underscores the dual importance of energy production and carbon allocation for biomass synthesis. Given the high protein cost for the EMP pathway, energy acquisition may be prioritized by anaerobes to withstand the fluctuating conditions. Our results suggest that microbial interactions with intercellular networks may play a critical role in facilitating the priming and subsequent degradation of sedimentary organic matter.

## 1. Introduction

Flat intertidal sediments are characterized by hydrodynamic conditions, resulting in the formation of various types of cohesive and permeable sediments. Microbial communities inhabiting these sediments exhibit adaptability to such fluctuating conditions, demonstrating metabolic flexibility both as generalists and specialists [1–3]. In coastal tidal flat, sandy sediments cover a larger area than mud sediments. Microbes within sandy sediments are particularly susceptible to disturbance caused by tidal and wave actions. Despite the relatively lean organic matter content in sandy intertidal sediments, microbial communities exhibit elevated metabolic activities, particularly in terms of CO_2_ production and biomass synthesis. This is attributed to the rapid turnover of seawater and the supply of labile organic matter [1,4–7]. Consequently, how the exogenous labile organic matter shapes the microbial metabolism and further alters indigenous sedimentary organic matter remains uncertain.

Investigating microbial metabolism is essential for understanding the early diagenesis of organic matter and its long-term burial in sedimentary systems. Microorganisms play a pivotal role in the transformation of sedimentary organic matter through a variety of metabolic pathways, which generally follow the redox cascade, involving oxygen, iron/manganese, nitrate/nitrite and sulfate reduction. However, when oxygen becomes depleted, fermentation is the primary process for cracking organic matter, subsequently providing volatile organic compounds (e.g., acetate, propionate) that support anaerobic processes [8]. Microbes in intertidal sediments have adapted to facultative and anaerobic strategies [8,9]. Sugars, primarily originating from phytoplankton, generally exhibit a more rapid turnover rate than other substrates, e.g., lipids and proteins [10]. Glucose is thus a commonly used substrate to stimulate microbial activity and metabolism, further gaining microbial processes of the labile organic matter [11,12]. In addition, glucose serves as an ideal component to study the priming effect of exogenous organic on the indigenous sedimentary organic matter [13–16].

Glucose metabolism is a well-defined process that involves upstream glycolytic pathways, such as the Embden-Meyerhof-Parnas (EMP), Pentose Phosphate (PP), and Entner-Doudoroff (ED) pathways, as well as the downstream tricarboxylic acid (TCA) cycle. These pathways are integral to central carbon metabolism pathways (CCMPs), which provide the foundation for carbon and energy metabolism for other biomolecules, such as proteins, lipids and carbohydrates. Moreover, the TCA cycle functions as a central energy engine for microbial growth. Although the three main glycolytic pathways share similarity in CCMPs, microbes tend to preferentially utilize specific pathways for catabolic processes. This preference can be characterized by microbial intercellular fluxes, which describe the distribution and direction of carbon flow through the CCMPs [17–20]. For instance, *Escherichia coli* and *Bacillus subtilis* metabolize glucose primarily through EMP and PP pathways with the flux ratios of 80% and 20%, respectively [21]. By contrast, *Pseudomonas* species (e.g., *P. putida*, *P. aeruginosa*, and *P. fluorescens*) predominantly utilize the ED pathway with over 90% flux directed through this pathway and only a minor flux into the PP pathway [22,23]. The large-scale studies of marine bacteria reveal a distinct metabolic flux pattern with 90% of strains using the ED pathway, compared to only 10% using the EMP pathway. However, the genome surveys suggest that higher ATP yield associated with the EMP pathway may be advantageous for anaerobes, whereas the ED pathway is more common among facultative anaerobes [24]. The apportionment of carbon flux through the CCMPs is hypothesized to be driven by energy supply and resistance to oxidative stress [24,25]. Therefore, further investigation into the flux patterns of microbial species, particularly anaerobes, is needed to deepen our understanding of microbial metabolic strategies.

Microbial intercellular flux can be estimated using position-specific ^13^C substrate incubation, a technique known as ^13^C-metabolic flux analysis techniques (^13^C-MFA), which has been widely used for growth optimization or specific substrate production in metabolic engineering [26,27]. The selection of position-specific ^13^C labelled glucose (e.g., glucose-1-^13^C or glucose-6-^13^C) is primarily driven by the carbon divergence on the CCMPs. Additionally, the concurrent application of different ^13^C labelled isotopologues enables the detailed tracing of intercellular fluxes in both individual microbes and microbial communities [8,18,20,28]. This approach has been employed to investigate intercellular flux patterns within environmental microbial communities, revealing diverse intercellular flux profiles under different conditions [18,19,29–31]. In soil systems, microbial communities predominantly use the PP pathway for glucose metabolism [19,32,33], which contrasts with the preference observed in pure strains. Detailed modelling analysis revealed equal importance for the ED and PP pathways [19,31]. In contrast, microbes in intertidal sediments favor the EMP as the dominant fermentation route to metabolize glucose [8]. Environmental stressors, e.g., water content, temperature, respiration inhibitor, oxygen level and even different growth times, can alter the flux pattern [8,30,31,34].

Metabolic flux patterns for environmental microorganisms are mainly estimated at the level of microbial communities, rather than the diversity at group or individual levels. Consequently, the behavior of specific microbial groups or individuals, as well as their interactions during glucose metabolism, remains poorly understood. To the best of our knowledge, Hutchinson et al. [8] first reported microbial flux patterns in intertidal sediments, identifying the dominance of the EMP pathway for glucose metabolism. These flux patterns exhibit significant variability depending on oxygen level and sampling time points. This raises the question of whether such variation is due to the rapid regulation of dominant microbial groups or the dynamic shifts in microbial groups or individuals with distinct flux patterns in response to environmental stressors. Studies of pure strains have demonstrated diverse flux patterns across different species, suggesting that exploring microbial groups or individuals could further elucidate the diversity of metabolic flux patterns within microbial communities. Additionally, functional genomic surveys could provide valuable insights into the regulatory mechanisms that govern these flux patterns.

To further probe the mechanism controlling the intercellular flux patterns of microbial communities in intertidal sediment, we conducted the incubation of intertidal sediment collected from the Nanhui tidal flat, East China Sea by using ^13^C-labelled glucose isotopologues. Combining the modelling ^13^C -MFA approach with the ^13^C-CO_2_ and PLFA production, this study aims: 1) to quantify the flux patterns across the microbial community and group levels; to further explore the relationship between functional genome and intercellular fluxes as well as functional metagenome abundance. This study is expected to provide insight into the intercellular dynamics of microbial groups or individuals, and further address microbial metabolism in the utilization of sedimentary carbon.

## 2. Materials and methods

### 2.1. Material and incubation setup

In April 2024, surface sediment (0−5 cm) was collected from the intertidal zone of Nanhui East Shoal, East China Sea (31.0213° N,121.9365°E) during low tide. The sediment was placed into sterile sampling bags. Additionally, 2 L of in-situ seawater was collected for further incubation. The sediment was mainly consisted of silt and sand with TOC amount ranging from 0.01–0.34 at% (with an average of 0.18wt%) [35].

Prior to incubation, the sediment was thoroughly homogenized. In-situ seawater was sterilized by filtration through a 0.22 µm membrane. A slurry mixture was then prepared by combining 12 g of wet sediment with 60 mL of seawater in a 100 mL wide-mouth bottle (measured volume of 140 mL). This operation was conducted in an anoxic glovebox. The headspace gas in each bottle was flushed with N₂ to keep anaerobic conditions following by the pre-incubation at 10°C for 3 days. Afterwards, seven types of ^13^C-labelled glucose isotopologues were added: D-glucose-1-^13^C, D-glucose-2-^13^C, D-glucose-3-^13^C, D-glucose-4-^13^C, D-glucose-5-^13^C, D-glucose-6-^13^C and D-glucose-U-^13^C_6_ (99%, Cambridge Isotope Laboratories, USA). Additionally, controls including unlabelled glucose, autoclaved samples, and no-glucose addition (Table 1). The sterilization step was to autoclave sediment at 120 ºC for 20 min. Each bottle was added with 1 mL of 6 mM glucose stock solution, resulting in a final concentration of approximately 100 μM in medium. For each treatment, three replicates were processed.

**Table 1:** Overview of sediment incubation with ^13^C-labelled glucose.

No.	Treatments	Label	Sediment (g)	Seawater (mL)	Replicates
1	Raw sediment	Raw	12.2	60	3
2	Sterilization	Autoclave	12.0	60	3
3	Glucose	Glc	12.5	60	3
4	Glucose-1-^13^C	C1	12.3	60	3
5	Glucose-2-^13^C	C2	12.1	60	3
6	Glucose-3-^13^C	C3	12.1	60	3
7	Glucose-4-^13^C	C4	12.2	60	3
8	Glucose-5-^13^C	C5	12.1	60	3
9	Glucose-6-^13^C	C6	12.2	60	3
10	Glucose-U-^13^C_6_	U	12.2	60	3

After glucose addition, the samples were incubated at 10 °C for 96 hours by monitoring the headspace CO_2_ concentration with the sampling time points at 6, 12, 24, 36, 48, 72, and 96 hours. At each interval, 10 mL of headspace gas was collected in aluminium gas bags, and 0.6 mL was promptly used to determine CO₂ concentration via GC-FID. Remaining CO_2_ gas at 72 hours was analysed for δ^13^C analysis. At the end of the incubation, slurries were centrifuged and the supernatants collected for further dissolved inorganic carbon (DIC) analysis. 0.5 g sediment of each replicate was collected and stored at −80°C for metagenomic analysis. DNA from different types of glucose were pooled for analysis. The remaining sediments were lyophilized for further analysis of phospholipid-derived fatty acids (PLFAs). The detailed analysis methods for CO₂ concentration, DIC and their carbon isotope composition are attached in the S1 File.

### 2.2. PLFA extraction and analysis

Five grams of lyophilized sediment were extracted in an ultrasonic bath with the modified Bligh-Dyer extraction method [4]. Prior to extraction, 5 µg of C_19_-PC (1,2-dinonadecanoyl-*sn*-glycero-3-phosphocholine) was added as an internal standard. Twenty mL of methanol: dichloromethane (DCM): phosphate buffer (pH 7.4; 2:1:0.8) was used following by sonication for 15 min and centrifugation at 3,500 rpm for 10 min. This step was repeated three times. The supernatant was combined and transferred to a separatory funnel following by the addition of DCM and water (1:1) for separation. The lower organic phase was collected and dried under N_2_. The total lipid extracts were purified on a silica column and eluted with 5 mL of DCM, acetone and methanol. The PLFA fraction in methanol was then saponified and esterified with sequential KOH (5%) and BBr_3_ (14%) in methanol solution. The yield fatty acid methyl esters (FAMEs) were analysed on gas-chromatography (GC), GC-mass spectrometer (GC-MS), and GC-isotope ratio-MS (GC-IRMS) for concentration and ^13^C analysis. The detailed methodology is attached in the S1 File.

### 2.3. DNA extraction and metagenome analysis

Sediment samples of 0.5 g were used for DNA extraction using the FastDNA® SPIN Kit for Soil (MP Biomedicals, US) following the manufacturer’s instructions. The DNA concentration and purity were measured on a Quantus Fluorometer (Picogreen), and integrity was assessed using 1% agarose gel electrophoresis. DNA extracts were fragmented to an average size of about 350 bp using Covaris M220 (Gene Company Limited, China) for paired-end library construction. Metagenome sequencing was performed on Illumina NovaSeq ™XPlus (Illumina, USA) sequencing platform (Shanghai Majorbio Biomedical Technology Co., Ltd.), the raw metagenomic sequencing data associated with this project have been deposited in the NCBI Short Read Archive database with accession number: PRJNA1245057.

Metagenome sequencing data were subjected to quality control. Adapter sequences at the 3’ and 5’ ends of reads were trimmed using FASTP [36] (https://github.com/OpenGene/fastp, v0.23.0). Low quality reads of less than 50 bp and average scores below 20 were removed to retain high-quality sequences. Contigs longer than 300 bp were selected from the quality-filtered reads for further processing. De novo assembly was performed using MEGAHIT [37] (https://github.com/voutcn/megahit, version 1.1.2). Open reading frames (ORFs) were predicted from assembled contigs using Prodigal [38] (https://github.com/hyattpd/Prodigal, v2.6.3). Only genes with a nucleic acid length of ≥ 100 bp were retained and translated into amino acid sequences. The predicted gene sequences of all samples were clustered using CD-HIT with a 90% identity threshold and 90% coverage [39] (http://weizhongli-lab.org/cd-hit/, version 4.6.1). The longest gene in each cluster was selected as representative sequence to construct non-redundant gene set.

High-quality reads were aligned to the non-redundant gene using SOAPaligner with a 95% identity threshold [40] (https://github.com/ShujiaHuang/SOAPaligner,v2.21). Gene abundance in each sample was calculated based on the alignment results. For taxonomic and functional annotation, amino acid sequences from non-redundant gene sets were compared against NCBI-NR, COG and KEGG databases using Diamond [41] (https://github.com/bbuchfink/diamond,version 2.0.13) with BLASTP (e-value of 1e-5). Taxonomic assignments were determined using the NCBI-NR library, and species abundances were calculated by summing the gene abundances assigned to each species. Functional annotation was performed by aligning to the EggNOG database with COG abundance calculated as the sum of the abundances of associated genes. Similarly, gene functions were annotated to KO, Pathway, EC and Modules using the KEGG database, and the abundances of each functional category were derived from the cumulative abundance of the corresponding genes.

### 2.4. Calculation method for ^13^C enrichment

Atomic Percent Excess (APE) of ^13^C-CO_2_ and PLFAs were calculated to compare ^13^C enrichment of different position-specific ^13^C labelled incubations.


$${\mathrm{\backslash [}}^{13}C\_APE\;{=\;}^{13}F_{tx}{\;-\;}^{13}F_{t0}$$ (1)

$${\mathrm{\backslash [}}^{13}F\;=\;\frac{13R}{{(}^{13}R+1)}$$ (2)

$$\begin{array}{c} 13R\;=\left(\frac{\delta 13C}{1000}+1\right)\;\;\times \;13\;R_{std} \end{array}$$ (3)

Where ^***13***^***F***_***t0***_ and ^***13***^***F***_***tx***_ represent the fractional abundance of ^13^C in the produced CO_2_ or PLFAs at start (*t*_*0*_) and at the time of sampling (*t*_*x*_), respectively. The value of ^***13***^***F***_***t0***_ was estimated using that from raw sediment at harvest by assuming no variation of ^13^C values of raw sediment during the incubation. ^*13*^*R* is the atom ratio of ^13^C/^12^C of CO_2_ or PLFAs and ^*13*^*R*_*std*_ is the standard value 0.011180 for VPDB.

The measured ^13^C-APE values from the six singly ^13^C-labelled glucose treatments were further normalized by their cumulative sum (Eq. 4). This normalization reflects the relative contribution of individual carbon atoms in glucose to the total ^13^C observed in the produced CO_2_ and PLFAs, compared to the uniformly ^13^C-labelled glucose. The normalized [^13^C-APE]_i_ values for each glucose carbon position, denoted as [^13^C-APE]_i_nor_, were subsequently used in the modelling flux analysis in Eq. 5 (Section 2.6).


$$\begin{array}{c} {\left[13C\_APE\right]}_{i\_nor}=\;\frac{{[}^{13}{C-APE]}_{i}\;}{\sum_{1}^{6}{{[}^{13}C-APE]}_{i}} \end{array}$$ (4)

Where [^13^C -APE]_i_ refers to the ^13^C-APE of CO_2_ or PLFA from incubations with six singly ^13^C labelled glucose isotopologues, where *i* denotes the position of the ^13^C label in glucose, e.g., Glc-1-^13^C, Glc-2-^13^C, Glc-3-^13^C etc.

### 2.5. Modelling ^13^C-metabolic flux analysis

The ^13^C-metabolic flux analysis was conducted on the modified model developed by Wu et al. [19,47]. The biochemical reactions are listed in S2 Table. In MFA analysis, the first reaction on the metabolic network (i.e., Glc → G6P) was assumed to 100 and other reactions were calculated relative to first reaction. The simulated ^13^C-CO_2_ or acetyl-CoA enrichment under six ^13^C-labelled glucose (as [^13^C-APE]_i _sim_) was used for the modelling optimization. The optimization objective is as in Eq. 5. In this study, the ^13^C-APE values of PLFAs were assumed to reflect those of the node acetyl-CoA in the CCMPs (S1 Table) given that acetyl-CoA is the precursor for the PLFA synthesis [42]. During modelling, the ^13^C-APE value vector of each PLFA was independently applied in the flux analysis. Additionally, microbially respirated CO_2_ by microbes was also used for metabolic flux analysis proposed by literature [18,43].


$${Obj=fmincon(\;\;\left[13C\_APE\right]}_{i\_nor}-{\left[13C_{APE}\right]}_{i_{sim}}\;\;)$$ (5)

Where [^13^C-APE]_i_nor_ is the ^13^C-APE values after normalization in Eq. 4. [^13^C-APE]_i _sim_ is the simulated values in the model. The optimization algorithm employed the non-linear optimization function *fmincon* built in MATLAB (2021, US). The model details were referred to work by Wu et al. [19,47] and the raw code in the *Supplementary data.*

## 3. Results

### 3.1. CO_2_ production during incubation

**Fig 1:**
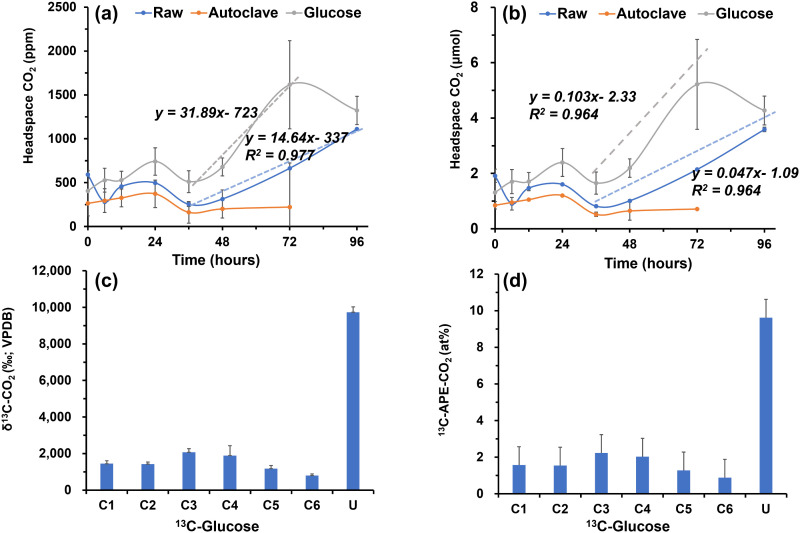
CO_2_ gas production during incubation and ^13^C-CO_2_ values at harvest. (a) CO_2_ concentrations (ppm) and (b) corresponding molar amount (μmol) in headspace were monitored over 96 hours of incubation. (c) δ^13^C and (d) atom percent excess of CO_2_ were measured from gas samples collected at 72 hours. Calculation based on Eq. 1-3. Label C1 ~ C6 and U represent the different ^13^C-lablled glucose isotopologues: glucose-1-^13^C, glucose-2-^13^C, glucose-3-^13^C, glucose-4-^13^C, glucose-5-^13^C, glucose-6-^13^C, and glucose-U-^13^C_6_, respectively.

Headspace CO₂ concentrations were monitored to assess microbial utilization of the amended glucose (Fig 1). During the initial 36 hours, no obvious CO₂ accumulation was observed. Subsequently, glucose-amended sediments exhibited markedly higher CO₂ production rates with an average increase rate of 31.9 ppm/h, equivalent to 0.103 μmol/h (Figs 1a and 1b). Raw sample also showed CO₂ production but at lower rates of 14.64 ppm/hour (eq. 0.0473 μmol/h). Autoclave control samples exhibited no detectable CO₂ production during the incubation period. After 96 hours, the CO_2_ level in the glucose treatments ceased to increase, and the incubation was then stopped for the following genome and chemical analysis.

At the end of incubation, the DIC concentration in the raw samples was 2.55 ± 0.37 mM on average, while it was much lower (1.60 ± 0.40 mM) in the treatment with natural glucose (S1 Table). In contrast, the ^13^C-glucose treatment showed a notably higher DIC concentration with an average of 2.95 ± 0.35 mM (S1 Table). The applied amount of glucose was identical, but the yielded CO_2_ varied, likely due to the sample heterogeneity. Excluding the natural glucose treatment, the excess DIC produced in the ^13^C-glucose treatment would be 0.40 ± 0.38 mM.

At harvest, headspace CO_2_ and DIC were sampled for δ^13^C analysis. The raw sediments exhibited δ^13^C-CO₂ values of −10.2 ± 1.3 ‰, while samples amended with glucose showed slightly more negative values of −13.2 ± 1.2 ‰. In contrast, substantial ^13^C enrichment was observed in the samples with ^13^C-glucose amendments. A strong correlation between δ^13^C values of gas CO_2_ and DIC was observed (S1 Fig). As expected, treatments with uniformly ^13^C-labeled glucose produced the highest δ^13^C-CO₂ values of up to 9730 ‰; whereas singly ^13^C -labeled glucose yielded δ^13^C-CO₂ values ranging from 800 to 2070 ‰ (Fig 1c). Correspondingly, ^13^C enrichment (expressed as ^13^C -APE) ranged from 0.88 to 2.23 at% with the sum of 9.53 at%, which was close to 9.62 at% observed for the uniformly ^13^C-glucose treatment (Fig 1d). One-way ANOVA revealed significant differences in δ^13^C and ^13^C-APE values (*p* = 0.011) among singly ^13^C-glucose treatments. The δ^13^C and ^13^C-APE values follow the order of ^13^C position in glucose: C3 ~ C4 > C1 ~ C2 > C5 > C6 (Fig 1).

### 3.2. ^13^C-PLFA concentrations

**Fig 2:**
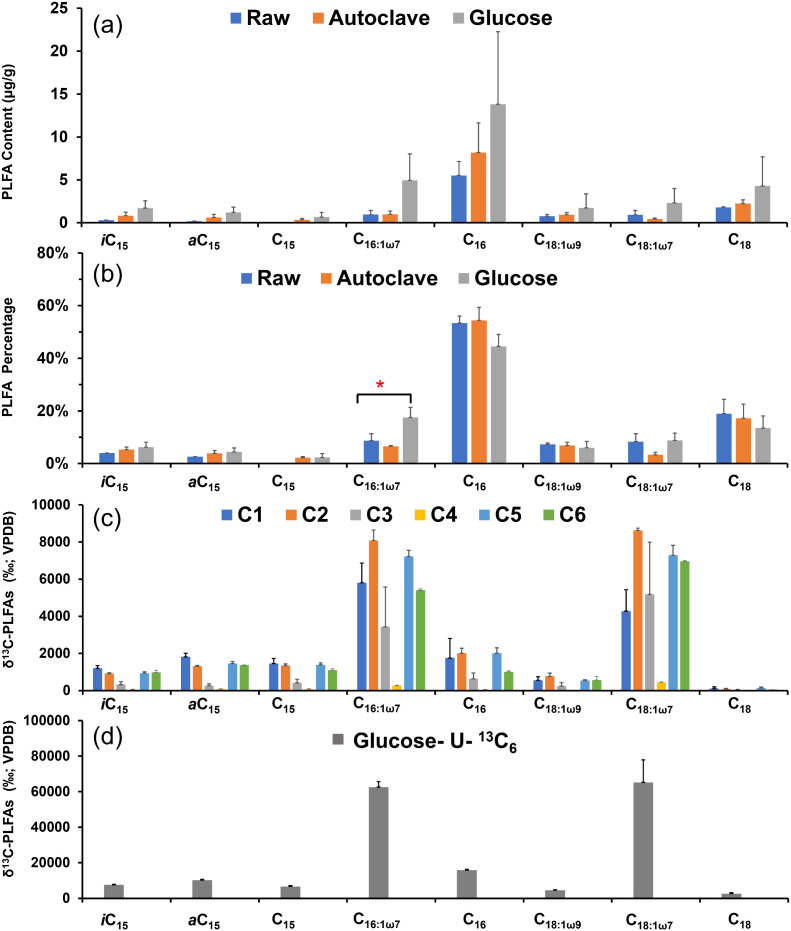
PLFA concentrations and carbon isotope enrichment after incubation with various ^13^C-glucose treatments at harvest. (a) PLFA concentrations (μg/g dry sediment) and (b) relative abundances (%) across treatments. (c) ^13^C composition (expressed as δ¹³C) of individual PLFAs in incubations with singly ^13^C-labelled and (d) uniformly ^13^C -labelled glucose treatments. Labels C1 to C6 refer to the treatments with six singly ^13^C-labelled glucose isotopologues.

The most abundant straight and unsaturated PLFAs in the sediment were C_16:0_, C_16:1ω7_, C_18:1ω9_, and C_18:1ω7_, with individual concentrations ranging from 0.2 to 15.7 μg/g. After incubation with glucose addition, the total PLFA concentrations exhibited an obvious increase by up to two-fold (Fig 2a). To account for potential sample heterogeneity (also observed in CO_2_ production; Fig 1), the relative abundance of PLFAs was also calculated. Notably, the relative proportion of C_16:1ω7_ increased markedly from 6.2% to 15.2% (Fig 2b).

For the treatment of ^13^C-labelled glucose, δ^13^C values of PLFA varied with the ^13^C position in glucose. As expected, the uniformly ^13^C-labelled glucose yielded the highest ^13^C-enrichment, particularly in C_16:1ω7_ and C_18:1ω7_, which reached δ^13^C values of up to 61,000 ‰ and 65,000 ‰, respectively (Fig 2d), corresponding to ^13^C-APE of 39.7 at% and 40.8 at%. Notably, even the singly labelled glucose treatments resulted in substantial ^13^C incorporation into these PLFAs. However, ^13^C-enrichment patterns were highly position-specific (Fig 2c). For *i*C_15_, *a*C_15_, C_15_, and C_18_, the ^13^C enrichment pattern followed the order of ^13^C position in glucose as: C1 > C5 ~ C6 > C2 > C3>> C4 (Fig 2c). In contrast, for C_16_, C_16:1ω7_ and C_18:1ω7,_ the order as: C2 > C5 > C6 ~ C1 > C3>> C4 (Fig 2c).

### 3.3. Modelling metabolic flux patterns

**Fig 3:**
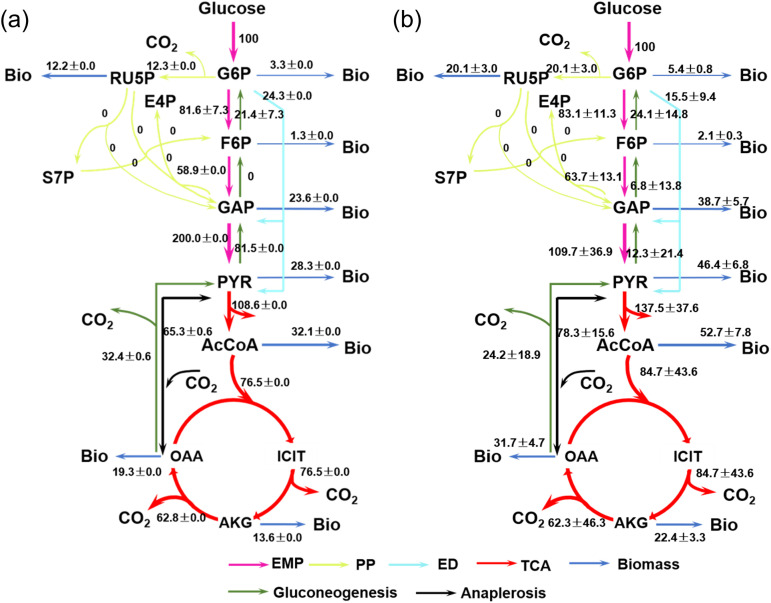
Intercellular metabolic flux through central carbon metabolism estimated by ^13^C-CO_2_ (a) and individual ^13^C-PLFA (b). The flux values for each biochemical reaction are proportional to the first step of glucose phosphorylation (Glucose -> Glucose-6-phosphate). The values are expressed as average and standard deviation.

The ^13^C-CO_2_ production was used to estimate the flux pattern for microbial communities in the sediment. At the community level, flux partitioning through CCMPs was estimated as 60.2% via EMP, 12.3% via PP, and 24.3% via ED pathways (Fig 3a). In contrast, ^13^C-MFA using individual ^13^C-PLFA profiles revealed the flux pattern of group- or species-level. The average fluxes across all PLFAs were 59.0% (EMP), 20.1% (PP), 15.5% (ED), consistently showing the EMP pathway as dominant but differing in PP and ED contributions. Notably, no net flux through the entire PP pathway was observed.

Carbon Use Efficiency (CUE), widely used in soil ecology, was also applied to estimate intercellular biochemical efficiency, following the assumption from Wu et al. [19,47] that excreted metabolites act as sink carbon in sediment. PLFA-based modelling indicated diverse CUE values across microbial groups, with the lowest CUE (i.e., 0.21; Table 2) found in producers producing *a*C_15_ and C_15_ PLFAs. These groups also exhibited extremely high fluxes through TCA with up to ca. 160% relative to glucose uptake, twice that observed in other microbial groups (Table 2).

**Table 2:** Microbial intercellular fluxes in the central carbon metabolism.

	Flux ratios relative to glucose uptake (%)				
Objectives^$^	EMP	PP	ED	TCA	CUE
*i*C_15_	59.4	21.9	18.7	54.4	0.73
*a*C_15_	74.9	16.3	8.7	159	0.21
C_15_	82.2	16.2	1.5	159	0.21
C_16_	68.7	21.9	9.4	60.6	0.70
C_18_	56.1	21.9	22.0	55.2	0.73
C_16:1ω7_	63.8	21.9	14.3	56.6	0.72
C_18:1ω9_	40.1	23.5	36.5	57.5	0.72
C_18:1ω7_	53.2	26.8	20.0	53.2	0.63
PLFA Mean	62.3	21.3	16.4	84.8	0.58
CO_2_	62.2	12.7	25.1	76.5	0.62

$ Objectives refer to the targets for optimization in ^13^C-metabolic flux analysis. PLFA Mean is the average flux ratios of ones based on the above ^13^C-PLFA. EMP, PP and ED are the upstream glycolytic pathways; TCA is a downstream tricarboxylic acid cycle. CUE is carbon use efficiency. CUE is the biochemical efficiency in the intercellular flux and is widely adopted in soil microbiology [19].

### 3.4. Metagenome characterizations

**Fig 4:**
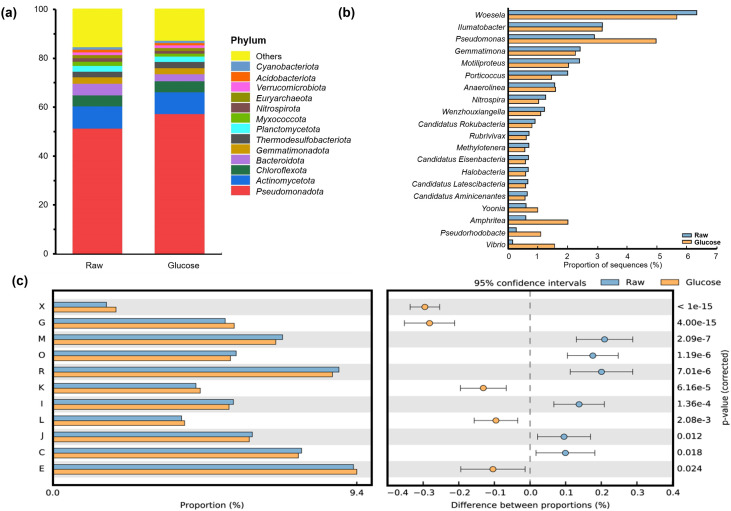
Microbial community composition and functional genomes after glucose amendment. (a) Taxonomic profiles at the phylum level and (b) genus level showing relative abundance in raw sediment and glucose-amended samples. (c) Glucose addition stimulates diverse functional genes annotated by the COG database. X: mobilome, prophages, transposons; G: carbohydrate transport and metabolism; M: cell wall/membrane/envelope biogenesis; O: posttranslational modification, protein turnover, chaperones; R: general function prediction only; K: transcription; I: lipid transport and metabolism; L: replication, recombination and repair; J: translation, ribosomal structure and biogenesis; C: energy production and conversion, E: amino acid transport and metabolism. Statistical significance was determined using STAMP with a *p*-value threshold of 0.05. Raw = Light blue, Glucose = Orange.

Gene annotation based on the NCBI-NR database illustrated the composition of the top 10 most abundant species across all samples, with low-abundance taxa grouped as others (Fig 4a). After the addition of glucose, microbial community composition structure did not obviously change, but the percentage slightly changed. For example, the percentage of *Pseudomonadota* increased from 51.3% to 57.2%, whereas the percentage of *Bacteroidota* declined from 4.7% to 2.9% (Fig 4a). At the genus level, *Woesela*, *Illumatobacter*, and *Pseudomonas* were the dominant groups in both treatments, of which the percentage showed significant change: percentages of *Woesela* decreased vs *Pseudomona* increased. In addition, the percentage of rare genus *Amphritea*, *Pseudomonas* and *Vibrio* increased substantially (Fig 4b).

**Fig 5:**
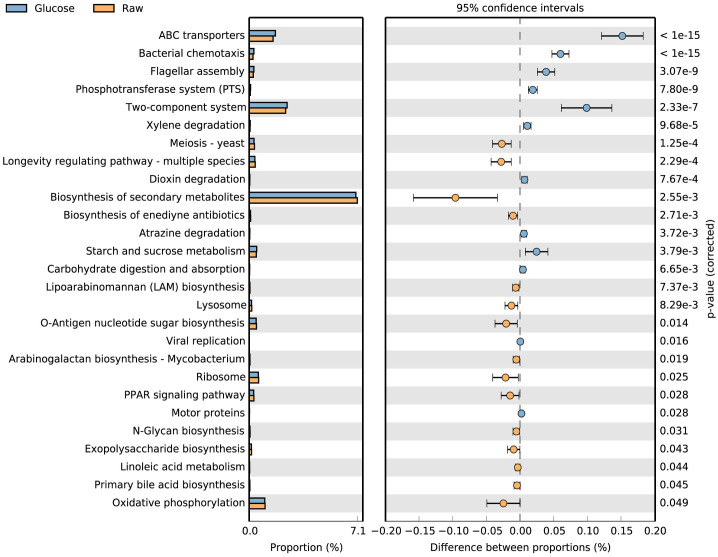
Microbial functional gene shifts in response to glucose addition based on KEGG annotation. Proportions of functional genes and mean proportional difference were performed using STAMP with a *p*-value threshold set to 0.05.

In addition to taxonomic composition, the functional genes of microbial communities showed further insights into microbial community responses to the glucose treatment. Based on COG database annotation, the addition of glucose stimulated the expression of mobilome (prophages, transposons; X), carbohydrate transport and metabolism, replication, recombination and repair (L), and amino acid transport and metabolism (E: Fig 4c). In contrast, KEGG-based functional annotation further showed that glucose enhanced activities in bacterial chemotaxis, ABC transporters, two-component system, and carbohydrate metabolism pathways (Fig 5). In particular, the pathway of biosynthesis of secondary metabolites was dominant, but the glucose addition did not appear to stimulate this process (Fig 5).

## 4. Discussion

### 4.1. Glucose priming effect for microbial communities

The artificial addition of glucose is intended to simulate the degradation of indigenous organic substrate on sedimentary organic matter. After the addition of glucose, substantial CO_2_ production and significant incorporation of ^13^C label into both CO_2_ and microbial biomass (PLFAs as an example) were observed. These findings indicate simultaneous activation of both anabolic and catabolic processes among sediment microbial communities, representing a critical component of secondary production in sediment [4,44]. However, the energy production rate, as inferred from the CO_2_ production curve, appeared relatively slow during the initial 36-hour monitoring (Fig 1). This contrasts with prior results from a ^14^C-glucose experiment, which showed fast energy release within hours or even minutes [11]. The discrepancy may be attributed to the equilibrium of dissolved inorganic and headspace after flushing the headspace for three days of pre-incubation, as well as to limitations in analytical sensitivity.

Moreover, the priming effect induced by glucose addition in this study appears to be transient, as CO₂ production plateaued after approximately 96 hours; whereas the untreated control samples exhibit a continued increase in CO₂ production beyond 72 hours, eventually reaching levels comparable to those observed in the glucose-amended treatments. The plateau of CO₂ production observed during glucose treatment likely reflects the limitation of nitrogen and phosphorus on cell growth and metabolism after the large addition of labile carbon [45,46]. In addition, this trend may imply an inhibitory effect of glucose-derived metabolites during incubation, such as the accumulation of volatile fatty acids, which are commonly detected in intertidal sediments [8]. Although volatile fatty acids were not directly measured in this study, metagenomic analysis reveals that the dominant functional pathway-biosynthesis of secondary metabolites- was downregulated by the addition of glucose (Fig 5). However, it requires further investigation into the effect of the potential glucose on the long-term incubation.

### 4.2. Anabolic activity in intertidal sediment

In addition to the respired CO_2_, glucose was also incorporated into newly synthesized microbial biomass, as evidenced by the production of ^13^C-PLFA. Notably, specific unsaturated PLFAs such as C_16:1ω7_, C_18ω9_, and C_18:1ω7_ exhibit a substantial increase in concentration. Despite inter-replicate variability, the relative abundance of PLFA C_16:1ω7_ increased significantly by 10% (Fig 2). When combined with community composition, this pattern suggests that C_16:1ω7_ is probably produced by *Pseduomodota,* which exhibits an increase in the gene abundance (Fig 3). This is further supported by the observation of C_16:1ω7_ in pure strains of *Pseduomodota* [47]. Total PLFA concentration rose from 4.2 μg g_dw_^-1^ to 12.4 μg g_dw_^-1^ after the glucose addition, corresponding to an increase of 46.7 μg C, which is relative to 10% of the applied glucose amount (6 μmol glucose eqv. 432 μg C; Detailed calculation sees S1 File). When considering bacterial-specific PLFAs such as C_15_, C_16:1ω7_, and C_18:1ω7_ in sediment [13], their concentration would be 2.53 ± 0.60 μg g_dw_^-1^. This translates to an estimated biomass carbon content of 838 ± 28 μg C (detailed calculation attached in S1 File), which is almost double the amount of glucose. This discrepancy suggests that exogenous glucose alone is insufficient to meet microbial C demands for biomass synthesis with extra contribution from indigenous sediment.

Together with the results from ^13^C-CO_2_ and ^13^C-PLFAs, these findings indicate that the glucose addition stimulates both catabolic and anabolic processes, both of which are supported by indigenous sedimentary carbon. These elevated energy requirements associated with these processes are further supported by the significant upregulation of ATP-binding cassette (ABC) transporter genes after the glucose addition (Fig 5) given their known role in ATP-dependent glucose transport and hydrolysis [48,49]. Additionally, genes associated with biosynthetic pathways for lipids and amino acids are also upregulated (Fig 4), further highlighting the increased carbon and energy demand for microbial biomass synthesis. Considering a low TOC in sandy tidal flat (ca. 0.18 wt%) [50], high C demand for benthic microbes suggests a high turnover efficiency, which is likely facilitated by hydrodynamic nutrient supply [4,8,13,51,52].

### 4.3. Diverse metabolic flux activities within microbial communities

The availability of diverse organic matter foster a variety of metabolic strategies within communities [1,9], which can be insighted from the distinct production patterns of ^13^C-CO_2_ and PLFAs under different ^13^C glucose isotopologues. This phenomenon is further associated with the intracellular fluxes observed on CCMPs observed in pure strains such as *E. coli*, *B. subtilis*, *P. putida* and *S. cerevisiae*, as well as in environmental communities [8,19].

In this study, we used ^13^C-CO_2_ and ^13^C-PLFAs independently to estimate intercellular fluxes through CCMPs. The former represents an average result at microbial community levels, while the latter provides insights at group and even individual levels. Based on the results of ^13^C-CO_2_, microbes in the sediment metabolize glucose through EMP, PP and ED with the flux ratios of 60.2%, 12.3%, and 24.3%, respectively. These findings are generally consistent with the average observations derived from ^13^C-PLFAs (Table 2). The dominance of EMP as the primary glycolytic route aligns with other studies at different sites [8]. However, Hutchinson et al. [8] found large variability in the EMP flux, ranging from 30% to 100%, across different intertidal sediments (muddy vs sandy), sampling points and incubation conditions (oxic vs anoxic). Such inconsistency might be attributed to different label strategies and applied models, which would result in mathematical differences in the flux estimation for specific microbes [20].

The overall pattern of microbial metabolic flux exhibits comparability across different sites when using identical models. When focusing solely on anoxic conditions as in this study, the estimated flux through EMP by Hutchinson et al. [8] remains relatively high at different time points. It remains debatable whether metabolic flux patterns are significantly altered by shifts in microbial community composition during incubation. However, our genomics data do not support this hypothesis, as microbial community structure showed no significant change after glucose treatment (Fig 3). Metabolic flux patterns are primarily governed by the functional genome [53]. In this study, we find that all three glycolytic pathways are complete (S2 Fig) with gene abundance of 56%, 25%, 19%, respectively. These ratios are remarkably consistent with metabolic flux estimates based on ^13^C-CO_2_ production, which yield 60.2%, 12.3%, 24.3% for EMP, PP and ED pathways, respectively. However, it should be acknowledged that gene abundance and metabolic fluxes are inherently distinct processes. Beyond genomic potential, intercellular fluxes are shaped by a complex interplay between enzymic regulation and environmental conditions [53,54]. Due to biochemical variability, metabolic fluxes can deviate from genomic predictions. For example, 1 mole of glucose yields two molar ATP via the EMP pathway while 1 molar ATP via the ED pathway in specific microbes. While EMP offers a higher energy yield, it typically operates more slowly than ED or PP, which may be advantageous for microbes with high energy demands but limited respiration rates under anoxic conditions. The observed similarity between genome abundance and metabolic flux ratios in this study may reflect an adaptive trade-off microbial community with balancing energy production efficiency and enzyme cost [24]. Altogether, the equivalence between gene abundance and metabolic flux distribution suggests that genomic regulation plays a role in shaping intracellular metabolic processes at the community level, at least under the conditions examined in this study.

To further elucidate the diversity of metabolic flux across different microbial taxa, we performed the ^13^C-MFA using individual ^13^C-PLFA, which provides taxonomical resolution. Across all bacterial groups, the EMP pathway is the dominant route to catalyze glucose with the fluxes ranging from 37.7% to 78%. In contrast, fluxes through PP pathways remain relatively constrained, ranging from 15.5% to 20.5%, whereas the ED pathway varies largely between 1.5% and 34.3%. These results indicate that both EMP and ED pathways play significant roles in microbial glucose metabolism in intertidal sediments. This observation is consistent with the studies on marine bacterial isolates [25]. However, a large proportion of marine bacterial strains appear to preferentially use ED over EMP [25], which reflects the metabolic adaptations of anaerobic microbial communities prevalent in intertidal sediments.

**Fig 6:**
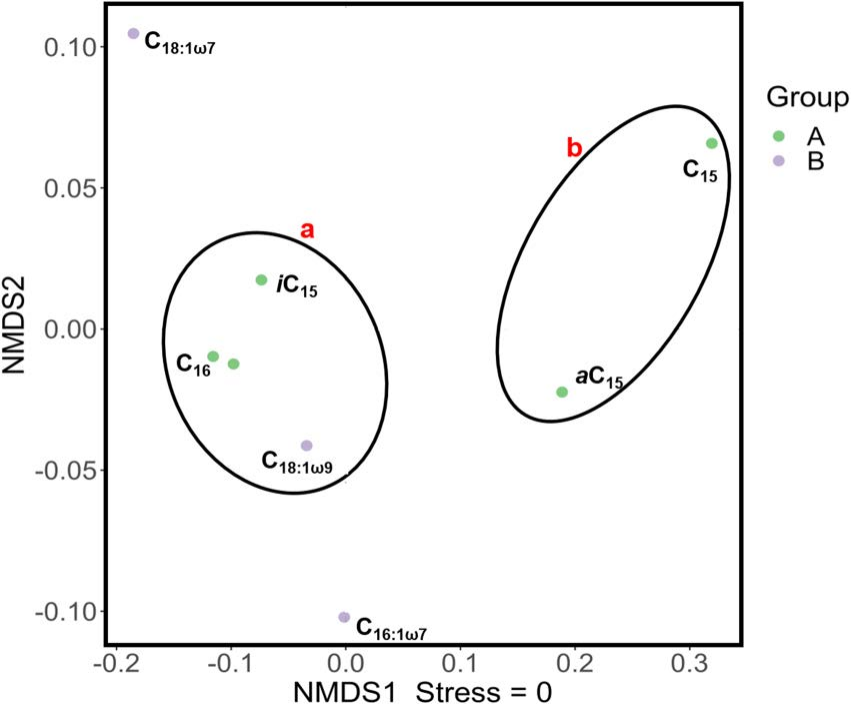
NMDS analysis of metabolic flux patterns for different bacterial groups based on PLFAs. Purple dots: unsaturated PLFAs; green dots: saturated PLFAs.

Furthermore, clustering of metabolic flux patterns across individual PLFAs revealed distinct pathway utilization among bacterial groups, particularly in their engagement of the EMP and ED pathways and downstream TCA cycle activities (Fig 6). Specifically, producers of *a*C_15_ and C_15_ PLFAs allocate 76% glucose through EMP, with the remaining fluxes distributed between the PP and ED pathways. A similar pattern was observed for C_16:1ω7_ producers, albeit with a notably higher flux directed into the TCA cycle. In marine sediments, branched and straight C_15_ PLFAs are likely contributed by sulfate reducers [55,56], which predominantly use acetate as carbon and energy source. The conversion of glucose into acetate may enhance TCA cycle activity in sulfate reducing bacteria, reflecting their metabolic preference and capacity for acetate oxidation. In contrast, bacterial producers of C_18:1ω9_ and C_18:1ω7_, commonly found among Gram-negative bacteria, demonstrate more balanced usage of three glycolytic pathways with equal flux distribution.

PLFA C_16:1ω7_ exhibits a prominent change in concentration after the addition of glucose. Together with microbial community data, this biomarker is likely derived from members of the phylum *Pseudomonadota* as previously demonstrated in pure strains [47,57]. In marine sediments, *Pseudomonadota* appear to channel ca. 60% of glucose through EMP. This contrasts with observation from pure strains of *Pseudomonas*, e.g., *P. putida*, *P. fluorescens*, which prefer to crack glucose via ED pathways accounting for over 90% of flux [22,23]. This apparent discrepancy may be attributed to metabolic interactions within the community. Notably, *Pseudomonas* species lack fructose-6-phosphatase, a key enzyme required for the forward operation of the EMP pathway. As a result, these organisms cannot efficiently utilize the EMP pathway unless fructose or other intermediates are externally supplied [22,23]. The elevated EMP flux observed in *Pseudomonas* in sediments could therefore result from cross-feeding, whereby metabolic metabolites are provided by co-occurring microbial taxa. Such cross-feeding interactions may play a crucial role in supporting microbial energy acquisition through a more ATP-efficient EMP pathway under anoxic conditions. This hypothesis is further suggested by genomic evidence, which shows upregulation of ABC transporter genes, which are energetically expensive systems responsible for the uptake of sugars and metabolites (Fig 4) [49]. Nevertheless, in the context of organic-lean intertidal sediment, anaerobic microbes appear to rely heavily on such cooperative metabolic networks to support both anabolic and catabolic processes. This interplay underscores the ecological importance of metabolic cross-feeding in optimizing energy yield and sustaining microbial activity via the EMP pathway, particularly under energy-limited conditions [24]. Considering the possibility of aerobic respiration, oxygen supply could stimulate energy production pathways and thereby regulate the biomass production including lipids [58]. A distinct pattern of ^13^C incorporation into fatty acids, similar to that observed in the soil system [19], would be expected under such conditions. However, this hypothesis requires further investigation in coastal sediment.

## 5. Conclusion

In this study, we investigated the microbial intercellular flux patterns in an intertidal sediment from the Nanhui tidal flat, East China Sea, by incubation with position-specific ^13^C labelled glucose. High production of ^13^C-CO_2_ and PLFAs relative to the supply of limited glucose indicates a positive priming effect for microbes to use indigenous sedimentary organic matter. Diverse ^13^C-PLFAs production patterns by different ^13^C in glucose confirm the differential microbial groups with distinct intercellular flux patterns within communities. The metabolic flux patterns on three main glycolytic pathways are mainly controlled by their functional genome abundance, suggesting balanced energy and protein cost for each pathway across the microbial community level. Modelling metabolic flux analysis reveals the dominance of the classic EMP pathway for microbes to use glucose for high energy yield. Metabolite cross-feeding may be an important mechanism for anaerobes to select the thermodynamically slow but high energy yield pathway EMP in marine sediments. This study provides an insight into the energy and cost input for microbes inhabiting intertidal sediments with position-specific ^13^C-labelling glucose, further addressing the role of microbial intercellular flux pathways for the understanding of quantifying the carbon fluxes in intertidal sediments.

## Supporting information

S1 File Instrumental methods for CO_2_ and PLFA analysis, and calculation method for priming effect. (DOCX)

S1 Table Concentration and ^13^C composition of DIC and CO_2_ in the end of incubation. (DOCX)

S2 Table The modelled biochemical ratios for EMP, PP, ED, TCA, gluconeogenesis, and anaplerotic reactions. (DOCX)

S1 Fig Correlation of carbon isotope between gas CO_2_ and dissolved inorganic carbon (DIC). (DOCX)

S2 Fig Reconstructed central carbon metabolism pathways based on the genome. (DOCX)
