# Supplementary material for: Glucose priming effect on microbial intercellular metabolic flux diversity in a marine intertidal sediment
Source: PLoS One. 2025 Nov 26;20(11):e0335053. doi: 10.1371/journal.pone.0335053 (PMC12654903; doi:10.1371/journal.pone.0335053)
Supplement: S1 File — (DOCX) [file pone.0335053.s001.docx]

**Supporting Information**

**S1 File*.* Instrumental methods for CO_2_ and PLFA analysis, and calculation method for priming effect**

***CO_2_ gas analysis***:

CO₂ was detected on GC-FID (GC 7890B, Agilent, USA). 0.5 mL headspace sample was injected at 120°C with split mode and then separated on a column of HP-PLOT Q PT (30 m * 0.32 mm; 20 μm in film thickness). The flow rate of carrier gas was 5 mL/min. The CO_2_ was converted into CH_4_ with a nickel catalyst. Detector temperatures were kept at 250°C. The temperature program for oven was 45°C for 30 min. The concentration was calibrated with three gas standards with 500, 1000 and 2000 ppm.

The carbon isotope composition of CO₂ (δ^13^C- CO₂) at harvest was performed on gas-chromatography-isotope ratio-mass spectrometer (GC-IRMS, Thermo Scientific, US). The column was same as the above concentration on GC-FID. The δ^13^C values are calibrated to the reference CO_2_ gas.

The concentration and carbon isotope dissolved inorganic carbon (DIC) at harvest were performed on GasBench connected to isotope ratio-mass spectrometer (Thermo Scientific, US). 200 μL phosphatic acid was added into 12 mL Exetainer vial (Labco, England) following by the flushing with helium on GasBench. Then 1 mL filtered medium was injected into the vials overnight. The yielded gas was introduced into the system for 8 times analysis. The δ^13^C values were calibrated to in-house carbonate standard with precision better than 0.5‰. The DIC concentration was calibrated to the freshly prepared bicarbonate solution with detect limit of above 0.1 mM.

***PLFA analysis***

PLFAs were quantified on GC- FID (GC 7890B, Agilent, USA). PLFAs were injected into the inlet at 300°C in splitless mode and separated on a column of HP-5ms (30 m * 0.25 mm; 0.25 μm in film thickness). The temperature program was: 70°C (1 min) increased to 130°C for 4 min at 20°C/min, then to 300°C at 4°C/min with holding for 10 min. Detector temperatures were keep at 250°C. The structure of PLFA was confirmed on GC-MS (GC 7890B and MS 5977A, Agilent, USA). Same oven program as the above GC-FID. MS source and quadruple temperatures were 230°C and 150°C, respectively. External mixed fatty acid methyl ester standard (37 FAMEs, CRM47885, Sigma) was used for structure verification.

The carbon isotope compositions (δ^13^C) of PLFAs were analyzed on gas-chromatography-isotope-ratio mass spectrometer (GC-IRMS, Thermo Scientific, USA). The PLFAs were separated on a column of TG-5MS (30 m * 0.25 mm; 0.25 µm film). The temperature program was 60°C (1min) increased to 130°C at 25°C/min, then to 250°C for 1 min at 3°C/min, followed by an increase to 300°C at 25°C/min for 10 min. The combustion temperature was 1000°C. The δ^13^C values relative to VPDB were calibrated to the mixed alkane standards with the precision of better than 1‰ (A7, purchased from Arndt Schimmelmann Group, Indiana University).

***Calculation method for glucose priming effect on CO_2_ and biomass production***

The DIC concentrations of raw and glucose treatment was respective 2.55 ± 0.37 mM vs 2.82 ± 0.78 mM with an increase of 0.26 ± 0.61 mM. However, we can see that the DIC concentration for natural glucose treatment was obviously lower than other ^13^C labelled glucose treatment. For glucose treatment, we used the ^13^C-glucose to compute CO_2_ production. The increasing concentration of DIC was 0.4 ± 0.38 mM whereas glucose concentration was 100 μM. The carbon recovery would be 0.4 ± 0.38/total concentration of 6 μmol to 60 mL medium, eqv. 100 μM. The carbon recovery would be 0.26 mM / 0.1 mM = 260%.

The total amount of PLFAs increased from 4.2 μg g_dw_^-1^ to 12.4 μg g_dw_^-1^ after the glucose addition. Given the carbon ratio of 74% (palmitate C_16_H_32_O_2_ as example), it is estimated that the added glucose (6 μmol eqv. 36 μmol Carbon) produces an extra 8.2 μg g_dw_^-1^* 0.74 * 7.7 g_dw_ = 46.7 μg carbon. The added glucose was 432 ug C, and glucose simulation effect would be ca 10%. If considering the specific bacterial PLFA in sediment, it would be C_15_, C_16:1ω7_ and C_18:1ω7_ ([Middelburg et al., 2000](#_ENREF_35)) of which the increased concentration for the three PLFAs would be 2.53 ± 0.60 g g_dw_^-1^, then converted biomass carbon would be 838 ± 28 μg C (0.035 g of carbon PLFA per gram of carbon biomass ([Middelburg et al., 2000](#_ENREF_35)) two times higher as the added glucose (100 μM*60 mL * 6 * 12 g/mol = 432 μg C)
