# Supplementary material for: Glucose priming effect on microbial intercellular metabolic flux diversity in a marine intertidal sediment
Source: PLoS One. 2025 Nov 26;20(11):e0335053. doi: 10.1371/journal.pone.0335053 (PMC12654903; doi:10.1371/journal.pone.0335053)
Supplement: S1 Table — (DOCX) [file pone.0335053.s002.docx]

**S1 Table. Concentration and ^13^C composition of DIC and CO_2_ in the end of incubation.**

| Treatment | DIC (mM) | ^13^C-DIC (at%) | CO_2_ (μmol) | ^13^C-CO_2_ (at%) |
| --- | --- | --- | --- | --- |
| Raw | 2.55 | 1.1193 | 2.14 | 1.0945 |
| auto | 1.75 | 1.1038 | 0.71 | 1.0946 |
| glucose | 1.60 | 1.1360 | 3.57 | 1.0912 |
| C1 | 3.16 | 3.0876 | 5.02 | 2.6618 |
| C2 | 2.86 | 2.7095 | 5.06 | 2.6328 |
| C3 | 2.94 | 2.8333 | 5.85 | 3.3215 |
| C4 | 2.82 | 3.3911 | 7.10 | 3.1223 |
| C5 | 2.33 | 1.8139 | 4.56 | 2.3746 |
| C6 | 3.05 | 2.4242 | 2.91 | 1.9729 |
| U | 3.48 | 10.5310 | 7.66 | 1.0946 |
