# Supplementary material for: Glucose priming effect on microbial intercellular metabolic flux diversity in a marine intertidal sediment
Source: PLoS One. 2025 Nov 26;20(11):e0335053. doi: 10.1371/journal.pone.0335053 (PMC12654903; doi:10.1371/journal.pone.0335053)
Supplement: S2 Table — (DOCX) [file pone.0335053.s003.docx]

**S2 Table. The modelled biochemical ratios for EMP, PP, ED, TCA, gluconeogenesis, and anaplerotic reactions. The detailed reactions and its mark numbers are below.**

| Pathways | Fluxes | Reaction* | Flux balance equation |
| --- | --- | --- | --- |
| *Glycolysis and gluconeogenesis* | *v_1_* | Glc (abcdef) → G6P (abcdef) | *v_1_ = 100* |
|  | *v_2_*_,_ *v_13_* | G6P (abcdef) ↔︎ F6P (abcdef) | *v_2_ = v_1_ + v_13_ – v_9_* – *v_18_ – br_1_* |
|  | v_3,_ *v_14_* | F6P (abcdef) ↔︎ GAP (cba) + GAP (def) | *v_3_ = v_2_ + v_14_ + v_11_+ v_12_ – v_13_ – br_2_* |
|  | *v_4_*, *v_15_* | GAP (abc) ↔︎ PYR (abc) | *v_4_ = 2v_3_ + v_15_ + v_12_ + v_10_ + v_18_ – v_11_ -v_14_ -br_3_* |
|  | *v_5_* | PYR (abc) → AcCoA (bc) + CO_2_ (a) | *v_5_ = v_4_ + v_17_ + v_18_ - v_15_ – v_16_ – br_4_* |
| *TCA cycle* | *v_6_* | AcCoA (ab) + OAA (ABCD) → ICIT (abABCD) | *v_6_ = v_5_– br_5_* |
|  | *v_7_* | ICIT (abcdef) → AKG (abdef) + CO_2_(c) | *v_7_ = v_6_* |
|  | *v_8_* | AKG (abcde) → OAA (abcd) + CO_2_(e) | *v_8_ = v_7_-br_6_* |
| *Pentose phosphate pathway* | *v_9_* | G6P (abcdef) → RU5P (bcdef) + CO_2_(a) | *v_9_ = v_9_* |
|  | *v_10_* | RU5P (abcde) + RU5P (abcde) → S7P (ababcde) + GAP (cde) | *v_10_ = 1/2*(v_9_ - v_12_ - br_8_)* |
|  | *v_11_* | S7P (abcdefg) + GAP (ABC) → F6P(abcABC) + E4P (defg) | *v_11_ = v_10_* |
|  | *v_12_* | RU5P (abcde) + E4P(ABCD) → F6P (abABCD) + GAP (cde) | *v_12_ = v_11_* |
| *Anaplerotic reactions* | *v_16_*, *v_17_* | PYR (abc) + CO_2_ (A) ↔︎ OAA (abcA) | *v_16_ = v_6_ + v_17_ + br_7_ -v_8_* |
| *Entner-Doudoroff Pathway* | *v_18_* | G6P(abcdef) --> PYR (abc) + GAP (def) | *v_12_ = v_11_* |
| *Biomass production* | *br_1_* | G6P → biomass | *br_1_* |
|  | *br_2_* | F6P → biomass | *^$^br_2_ = α_1_*br_1_* |
|  | *br_3_* | GAP → biomass | *^$^br_3_ = α_2_*br_1_* |
|  | *br_4_* | PYR → biomass | *^$^br_4_ = α_3_*br_1_* |
|  | *br_5_* | AcCoA → biomass (e.g., fatty acids) | *^$^br_5_ = α_4_*br_1_* |
|  | *br_6_* | AKG → biomass | *^$^br_6_ = α_5_*br_1_* |
|  | *br_7_* | OAA → biomass | *^$^br_7_ = α_6_*br_1_* |
|  | *br_8_* | RU5P → biomass | *^$^br_8_ = α_7_*br_1_* |

*The letters in brackets after metabolite refer to the order of C atoms.

^$^Biomass production rates *br_2_*, *br_3_*, *br_4_*, *br_5_*, *br_6_*, *br_7_* and *br_8_* are constant relative to *br_1_,* and the values are [0.39 7.15 8.58 9.74 4.14 5.86 3.71] for modelling metabolic flux analysis, which are adapted from Wu et al. (2022, 2022) by assuming the ratio of Gram-positive and Gram-negative bacteria to be 1:1.
