# Supplementary material for: Glucose priming effect on microbial intercellular metabolic flux diversity in a marine intertidal sediment
Source: PLoS One. 2025 Nov 26;20(11):e0335053. doi: 10.1371/journal.pone.0335053 (PMC12654903; doi:10.1371/journal.pone.0335053)
Supplement: S1 Fig — (DOCX) [file pone.0335053.s004.docx]

**S1 Fig. Correlation of carbon isotope between gas CO_2_ and dissolved inorganic carbon (DIC).**
