# Supplementary figures and images for: Glucose priming effect on microbial intercellular metabolic flux diversity in a marine intertidal sediment
Source: PLoS One. 2025 Nov 26;20(11):e0335053. doi: 10.1371/journal.pone.0335053 (PMC12654903; doi:10.1371/journal.pone.0335053)

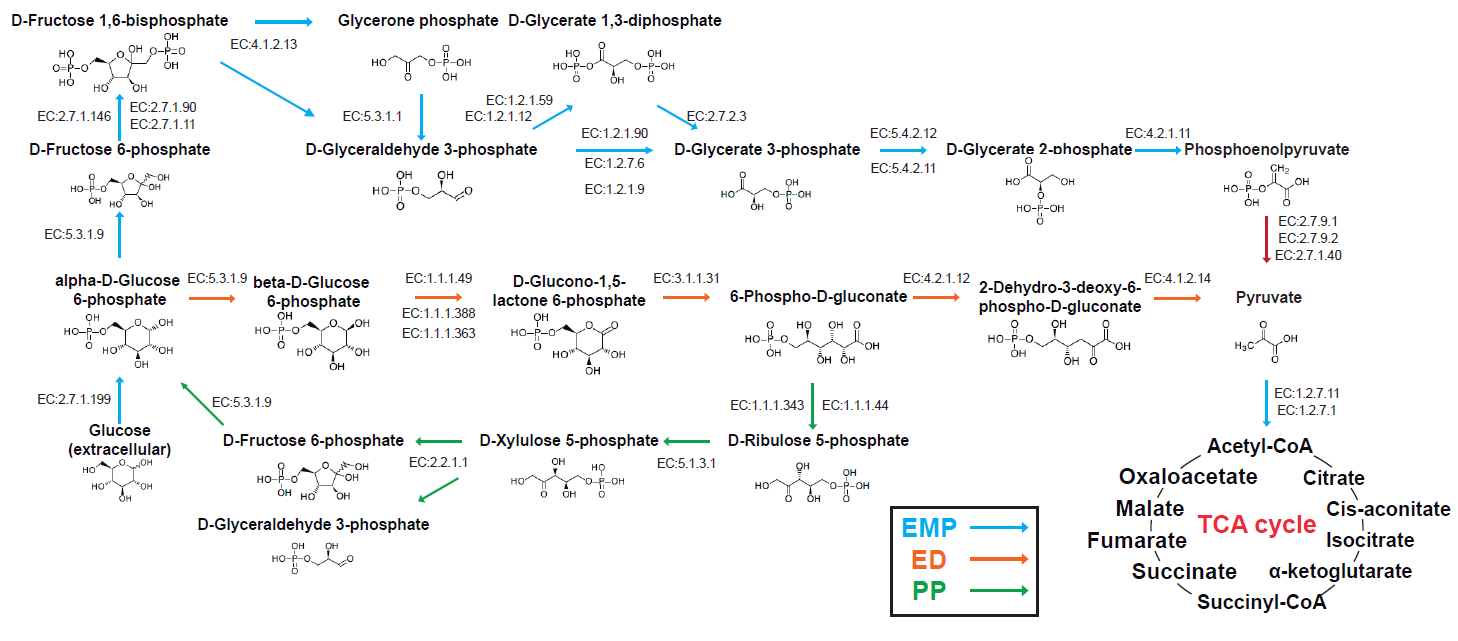


**S2 Fig. Reconstructed central carbon metabolism pathways based on the genome.**

Supplement: S2 Fig — (DOCX) [file pone.0335053.s005.docx]
